# Assessing suboptimal health status in the Saudi population: Translation and validation of the SHSQ-25 questionnaire

**DOI:** 10.7189/jogh.14.04030

**Published:** 2024-02-02

**Authors:** Mohamed Ali Alzain, Collins Otieno Asweto, Sehar-un-Nisa Hassan, Mohammed Elshiekh Saeed, Ahmed Kassar, Kamal Elbssir Mohammed Ali, Mouna Ghorbel, Rafat Zrieq, Bandar Alsaif, Wei Wang

**Affiliations:** 1 Department of Public Health, College of Public Health and Health Informatics, University of Ha’il, Ha’il City, Saudi Arabia; 2 Department of Community Medicine, Faculty of Medicine and Health Sciences, University of Dongola, Dongola, Sudan; 3 Department of Community Health, School of Nursing, University of Embu, Embu, Kenya; 4 Faculty of Medicine, National University-Sudan, Khartoum, Sudan; 5 Department of Physiology, Faculty of Medicine, University of Dongola, Dongola, Sudan; 6 Department of Community Health, Occupation Health and Safety Program, Northern Boarder University, Arar, Saudi Arabia; 7 Department of Biology, College of Sciences, University of Hail, P.O., Ha’il City, Saudi Arabia; 8 Applied Science Research Centre, Applied Science Private University, Amman, Jordan; 9 Clinical Research Center, First Affiliated Hospital, Shantou University Medical College, Shantou, China; 10 Centre for Precision Health, Edith Cowan University, Joondalup, Western Australia, Australia; 11 On behalf of Global Health Epidemiology Research Group (GHERG) & Global Suboptimal Health Consortium (GSHC)

## Abstract

**Background:**

Suboptimal Health Status (SHS) is realised as a vital feature for improving global health. However, the Arabian world does not have a validated instrument for screening SHS in their population. Therefore, the study aimed to evaluate the psychometric properties of Arabic-translated SHS (ASHSQ-25) in the Saudi Arabian population.

**Methods:**

We conducted a cross-sectional study among the conveniently sampled 1590 participants from the Saudi population (with a 97.4% response rate). The data was gathered through an online survey and then exported into SPSS and AMOS version 26.0 for analysis. Mann-Whitney and Kruskal-Wallis tests were used to identify the median difference between demographic groups. The one-tailed 90% upper limit of SHS scores was chosen as the cut-off criteria for SHS. Reliability and confirmatory analysis were performed for the psychometric evaluation of ASHSQ-25 in the Saudi Arabian context.

**Results:**

This study demonstrates that the ASHSQ-25 has good internal consistency, interclass correlation coefficient (ICC) = 0.92; 95% confidence interval (CI) = 0.91–0.93) and reliability (Cronbach’s α = 0.92). The confirmatory factor analysis (CFA) results indicated a good fit of the databased on the CMIN/degrees of freedom (df) = 4.461, comparative fit index (CFI) = 0.94, Tucker Lewis index (TLI) = 0.93, and Root Mean Square Error of Approximation (RMSEA) = 0.05. The result factor loadings for each item were high (≥ 0.55), except for one item from the immune system subscale. The SHS cut-off point for ASHSQ-25 was 33, leading to a 23.7% prevalence of SHS.

**Conclusions:**

This study reveals that ASHSQ-25 has appropriate internal consistency and structural validity to assess SHS in an Arabic-speaking population; therefore, it is recommended.

Suboptimal Health Status (SHS), commonly characterised as a low-quality health state [1], is an intermediate state between ideal health and a diagnosable illness; is indicated by decreased energy, physiological function and self-adaptation ability, which does not mean suffering from any disease [1,2]. It is recognised as a vital feature of prevention medicine and is, therefore, a construct of great interest to be measured in the field of Public Health [1–3]. The conceptualisation of SHS has its roots in Traditional Chinese Medicine (TCM), ascribed to lower levels of physical, psychological, and social adaptation and functioning of individuals that inhibit them from experiencing thriving lives. [3,4]. The distinguishing attributes of SHS are its reversibility and bidirectionality [4,5]; thus, accurately identifying individuals with SHS has a significant role in designing appropriate public health interventions and has wide applications to promote community health [5]. In the past few years, both objective methods (physiological and biochemical tests) [6] and subjective measures (clinician-administered and self-report scales) [7–10] have been developed to measure SHS. Suboptimal Health Status Questionnaire-25 (SHSQ-25) can transform the subjective manifestations of SHS in a relatively objective way depicted through scores on various domains of sub-health [1,2,10,11].

The SHSQ-25 encompasses five domains with 25 items [10]. These five domains include fatigue, cardiovascular system, digestive tract, immune system, and mental status. SHSQ-25 has gained increased popularity in the past few years and has been used by researchers to measure sub-health status in various studies [12–15]. However, at present, it has been assessed for its psychometric properties in limited regions of the world, translated into a few languages [5]. It has been validated in Chinese [10], Korean [16], Iran [14], Russian [17], and Ghanaian [18]. The first evidence about the reliability, validity, and factor structure of SHSQ-25 came from a large-scale study conducted in China [10]. Other study was conducted on the Ghanaian population in a sample of 263 individuals [19]. The invariance analysis supported a three-domain model – fatigue, immune-cardiovascular system, mental status rather than the five-domain model and the internal consistency values for these three domains were > 0.81. However, this study used the English version of the scale and authors of this study warranted the need for translation from English to the local Ghanaian language [18]. They acknowledged that a significant number of the participants were not well-versed in the English language used in the questionnaire, which might impact the responses on self-report SHSQ-25. Two studies used the Russian version of SHSQ-25; first reported the relationship of SHS with endothelial dysfunction [19], and the second demonstrated the impact of low physical activity on SHS [17] but did not report on the psychometric analysis of SHSQ-25.

A recent study completed the translation of SHSQ-25 into the Korean language and evaluated its psychometric properties by administering the scale to 460 participants recruited from one centre in Korea [16]. Findings demonstrated adequate reliability and validity to support the original five-factor multidimensional structure for SHSQ-25. This study contributed to the literature by developing the Korean version of the scale and additional evidence about the reliability, validity, and factor structure of SHSQ-25. Thus, a review of the current literature shows notorious gaps due to the non-availability of standardised and valid SHSQ-25 in Arabic to measure SHS [5].

SHS is a significant risk factor for chronic diseases. Previous research demonstrated that SHSQ-25 had shown promising results in the early detection of high risk-populations and thus substantially contributed to reducing disease burden through the implementation of preventive interventions [20,21]. A systematic review of research demonstrated a high prevalence of chronic health conditions among the Saudi population and its implications on the household economic burden and nation’s economy [22]. The country is on its way to achieving socioeconomic development and population health goals under the Vision 2030 [23]. The Saudi government is committed to taking several steps to pursue better health and quality of life for people living in Kingdom of Saudi Arabia (KSA). An accurate assessment of SHS can identify individuals vulnerable to developing noncommunicable diseases (NCDs) [24].

At present, the Arabic language has no self-report SHS tools available for utilising and conducting such assessments. Thus, translation, adaptation, and cross-cultural validation of SHS tools are warranted to achieve the goals of community health assessment. Arabic is the national language, widely spoken and understood by most of the population in Saudi Arabia. To overcome barriers associated with limited verbal fluency in the English language in KSA and to expand the use of the SHSQ-25 to measure SHS at a population level in various other Arabic countries, it is essential to translate the SHSQ-25 linguistically and cross-culturally into Arabic language. Subsequently, validate the Arabic version of SHSQ-25 (ASHSQ-25) in the Saudi population. The current study, therefore, aims to translate the SHSQ-25 into Arabic language and determine the reliability, validity, and factor structure of the ASHSQ-25 in Saudi populations.

## METHODS

### Study design, population, and sampling procedure

A cross-sectional study was conducted among adult populations in KSA. An online survey was carried out from 15 May to 5 June 2022. The online questionnaire was shared through Twitter and WhatsApp, with respondents selected via a convenient sampling method. Focal persons were selected from different regions to collect data in a diversified manner and recruit participants. Questionnaires were also distributed at different times and dates to recruit more participants. Eligible respondents read the informed consent and then filled in the questionnaire.

### Sample size determination

Using Yamane’s formula [25],



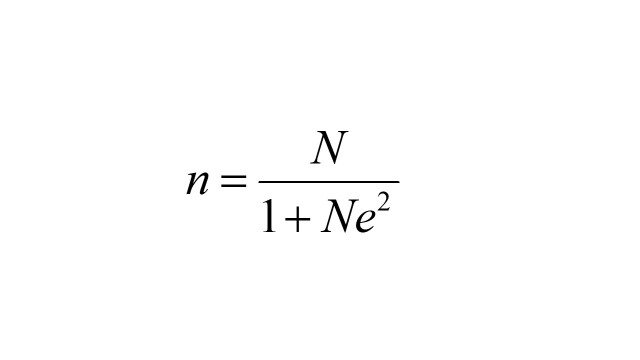



with allowing a +/− 3% margin error and given total target population (≥18 years) in the KSA is 22 9216 29 [26].



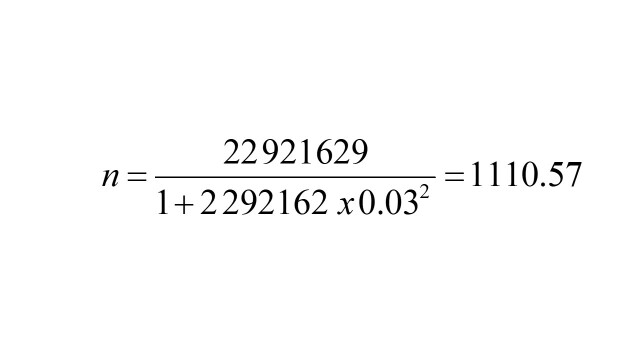



A minimum sample size was increased by 10% to account for non-response, given 1221 participants.

### Inclusion and exclusion criteria

The following were considered as inclusion criteria: 1) no previous experience with psychiatric or somatic illnesses; 2) aged ≥ 18; and 3) not been on medication for a fortnight prior. In addition, people with abnormalities such as diabetes, cardiovascular, respiratory, genitourinary, digestive, or circulatory disease were excluded.

### Measurement of suboptimal health status

The SHSQ-25 consists of five domains with 25 items, as detailed by Yan et al. [10]. Each item is graded on a 5-point Likert-type scale from never to always (0–4), and the computed sum of the 25 items' scores gives the SHSQ-25 score [27].

### Translation

**Figure 1:**
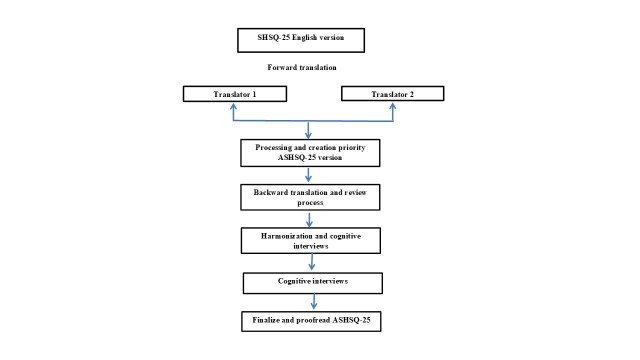
Flowchart for process of translation.

The SHSQ-25 was converted to Arabic language according to the WHO’s Guidelines on Instrument Translation to guarantee language and cultural similarity [27]. **Figure 1** shows the process of the translation flowchart. Two independent researchers conducted the actual forward SHSQ-25 translation from English to Arabic, proficient in both Arabic and English and conversant with the SHSQ-25's constructs. After consensus between the two translators and additional checking by the senior authors, the two translated versions were eventually combined into a single Arabic version. The other two bilingual English-Arabic translators, who had no prior knowledge SHSQ-25, then translated the concorded of the SHSQ-25 Arabic version back into English. The original and back-translated versions were then examined to guarantee linguistic, cultural, and conceptual similarity.

Cognitive interviews were performed to assess the cultural suitability, clarity, acceptability, and adequacy of the translated points in the SHSQ-25 Arabic version. Adjustments were made to the translated SHSQ-25 Arabic version using input from the cognitive interviews. Then, more interviews were conducted to look at the changes. Grammar, spelling, and formatting errors were checked. The ASHSQ-25 is presented in Appendix S1 in the **Online Supplementary Document**.

### Ethical considerations

The Scientific Research Ethics Committee at the University of Hail reviewed and approved the project proposal after reviewing all relevant documents, including the research questionnaire. The committee issued the ethical approval letter (H-2021-221) dated 6 November 2021.

Study procedures ensured the implementation of relevant ethical principles and obtaining informed consent. The consent form explained the purpose of the study, voluntary participation, the right to withdraw at any stage, and possible risks and benefits gained from participation in the study. Moreover, data anonymity was maintained during all stages of research, and no identifying information was collected. Researchers ensured the confidentiality of participants' data security and restricted the access to data files to authorised persons. The cumulative analysis of responses is completed in accordance with the objectives of the research and to draw inferences for study implications.

### Statistical analyses

We performed non-parametric tests (Mann-Whitney and Kruskal-Wallis) to determine the difference between groups because the normality test of the SHS score was violated. For the determination of the cut-off point for Arabic-SHSQ-25, the 90% upper limit of SHSQ-25 scores with one tail was utilised as the SHS cut-off point [28].

The reliability within different domains and item internal consistency (IIC) was measured. The internal consistency was analysed by computing Cronbach's α coefficient, which ranges from 0 to 1. Exceptional (≥ 0.9), noble (0.8–0.9), allowable (0.7–0.8), problematic (0.6–0.7), poor (0.5–0.6), and unacceptable (≤ 0.5) were the range of Cronbach's α values. An IIC was proposed to be supported by Cronbach's α of 0.40 [29].

Structural validity is the extent to which an instrument's scores accurately represent the dimensionality of the construct to be measured [30]. Confirmatory factor analysis (CFA) was utilised to evaluate the structural validity of the Arabic SHSQ-25 scale's measurement model. The generalise weighted least squares method was used in CFA because it is appropriate for ordinal data. A correlated 5-factor model was explored. The χ^2^ root mean square error of approximation (RMSEA), goodness-of-fit index (GFI), and adjusted goodness-of-fit index (AGFI) were used to evaluate the model's goodness of fit while performing maximum likelihood factor analysis and Promax rotation. RMSEA < 0.08, GFI and AGFI > 0.90, and CMIN/df value < 2 was considered a good fit, whereas a value < 5 was an acceptable fit [31,32]. By using Pearson correlation analysis, the associations between the various questionnaire domains (fatigue, cardiovascular health, digestive tract, immune system, and mental health) were examined. SPSS version 26.0 for Windows (IBM Corp., Armonk, NY) was used for statistical analyses CFAs were carried out using AMOS version 26.0 for Windows (IBM Corp., Armonk, NY). For all analysis of *P*-values, a two-tailed *P* < 0.05 was considered significant.

## RESULTS

### Sociodemographic characteristics of respondents

The total number of participants in this survey included 1590, males accounting for 67.6%, and nearly all (95.3%) respondents were Saudi nationality. Almost half of them (46.3%) were in the age group between 20–30 years, followed by 26.4% in the age group between 31–40 years. Almost two-thirds (61.5%) of respondents had a college degree, followed by more than a quarter (25.5%) completed high school. Subsequently, more than half of the participants were employees (53.9%) either in the health sector (20.4%) or non-health sector (33.5%), while student participants account for 23.3%. The median score of SHS significantly decreases as age increase. Subsequently, it was higher in females, those widows and divorced, and the occupation as Self-employed, student, and unemployed than their corresponding targeted counterparts, as mentioned in **Table 1**.

**Table 1:** Demographic factors of the study population

Variables	n	%	Median (IQR)	*P-*value
**Age**				
<20 y	133	8.4%	25 (28.5)	<0.001*
20–30 y	736	46.3%	22 (20)	
31–40 y	420	26.4%	20 (18)	
41–50 y	240	15.1%	17 (17.5)	
>50 y	61	3.8%	15 (15.5)	
**Gender**				
Male	1075	67.6%	19 (18)	<0.001†
Female	515	32.4%	25 (21)	
**Marital status**				
Married	746	46.9%	18 (18)	<0.001*
Single	796	50.1%	22 (21)	
Widow	9	0.6%	28 (26)	
Divorced	39	2.5%	26 (20)	
**Education level**				
Illiterate	2	0.1%	16 (0)	0.123*
Primary school	8	0.5%	14.5 (5)	
Middle school	28	1.8%	16 (27.5)	
High school	406	25.5%	22 (25)	
Graduate	978	61.5%	20 (18)	
Postgraduate	168	10.6%	19.5 (19)	
**Occupation**				
Employee in non-health sectors	532	33.5%	20 (19)	<0.001*
Employee in health sectors	324	20.4%	18 (19)	
Labourer	16	1.0%	15 (19.25)	
Self-employed	52	3.3%	23 (21.5)	
Student	370	23.3%	23 (23.5)	
Housewife	102	6.4%	20 (19.25)	
Retired	26	1.6%	15.5 (9.75)	
Unemployed	168	10.6%	22 (20)	
**Nationality**				
Saudi	1515	95.3%	20 (20)	0.857†
Non-Saudi	75	4.7%	19 (23)	

IQR – interquartile range

*Mann-Whitney test.

†Kruskal-Wallis test.

### Reliability analysis

The Cronbach’s α of ASHSQ-25 was 0.918, demonstrating high reliability (greater than 0.70 as recommended). **Table 2** demonstrates the internal consistency of subject domains and composite scores. The IIC for SHSQ-25 four domains (fatigue, mental health, cardiovascular, and digestive) ranged from 0.49 to 0.96, revealing that all items fulfilled the standard of good internal consistency.

**Table 2:** Reliability analysis of Arabic version of SHSQ-25

Item	No. of questions	Cronbach's α	IIC	ICC (95% CI)
Fatigue	9	0.80	(0.68–0.95)	0.79 (0.78–0.81)
Mental health	7	0.81	(0.49–0.92)	0.79 (0.77–0.80)
Cardiovascular	3	0.67	(0.85–0.90)	0.67 (0.64–0.70)
Digestive	3	0.82	(0.75–0.96)	0.81 (0.79–0.83)
Immunity	3	0.65	(0.62–0.95)	0.65 (0.62–0.68)
Total	25	0.92	–	0.92 (0.91–0.93)

ICC – internal consistency, IIC – item internal consistency, SHSQ-25 – Suboptimal Health Status Questionnaire-25

### Structural validity

**Figure 2:**
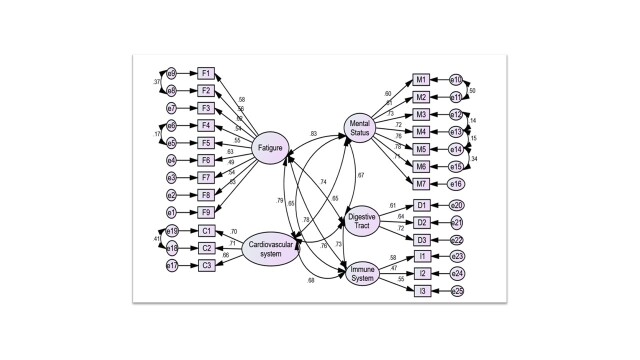
Structural equation analysis shows Suboptimal Health Status Questionnaire-25 (SHSQ-25) instrument's five-domain structure as represented by a confirmatory factor model that displays the standard factor loadings. Fatigue, cardiovascular system, mental health, digestive system and immune system demonstrated good-moderate reliability.

CFA was used to investigate the internal factor structure of the questionnaire. The Kaiser-Meyer-Olkin measure of sampling adequacy was 0.94, and Bartlett’s test of sphericity was significant (ᵡ^2^ = 15530.78, *P* < 0.001), suggesting that CFA was appropriate. The fit indices for the model shown in **Figure 2** fell within the acceptable range: CMIN/df = 4.46, CFI = 0.94, TLI = 0.93, and RMSEA = 0.05. Five factors were included in the 25 items. To achieve model fitness, correlations between errors (EC) for the 7-item pairs with the highest modiﬁcation indices (item F1-item F2, item F4-item F5, item C1-item C2, item M1-item M2, item M3-item M4, item M4-item M5, and item M5-item M6) were added.

**Table 3** displays the standardised correlation coefficients between variables and factors. The resulting factor loadings for each variable were high. The indicators have loadings that equal or surpass 0.55, except for one item from the immune system subscale. All indicators were statistically significant (*P* > 0.01). Item F2 has the lowest loading in the subscale immune system with a value of 0.47. The highest score is 0.78, which was obtained for item M6 in the subscale mental status. The acquired results support the ﬁve-factor structure proposed for the instrument and thus provide robust evidence of the model's construct validity. In addition to the ﬁve common factors, a final model which includes correlations between errors (R) but only for the seven-item pairs with the highest modiﬁcation indices (item F1-item F2, item F4-item F5, item C1-item C2, item M1-item M2, item M3-item M4, item M4-item M5, and item M5-item M6) was added (**Table 3)**. The pathways between domains and their variables are demonstrated in **Figure 2**.

**Table 3:** Factor loadings for the ﬁve-factor model, including correlations between error terms

Factor	Item	Standardised estimates	t-value	*P-*value	Factor loading	Error term R
Fatigue	F1	0.53	14.80	<0.001	0.58	0.37
	F2	0.54	16.48	<0.001	0.56	
	F3	0.49	15.52	<0.001	0.62	
	F4	0.63	18.20	<0.001	0.54	0.17
	F5	0.55	16.65	<0.001	0.55	
	F6	0.54	16.38	<0.001	0.63	
	F7	0.62	17.96	<0.001	0.49	
	F8	0.56	16.85	<0.001	0.54	
	F9	0.58	17.27	<0.001	0.53	
Mental	M1	0.60	20.47	<0.001	0.60	0.50
	M2	0.63	28.13	<0.001	0.61	
	M3	0.73	22.33	<0.001	0.73	0.14
	M4	0.72	21.89	<0.001	0.72	0.14/0.15
	M5	0.76	22.75	<0.001	0.76	0.15/0.34
	M6	0.78	23.28	<0.001	0.78	0.34
	M7	0.72	22.09	<0.001	0.71	
Cardiovascular system	C1	0.66	23.78	<0.001	0.70	0.41
	C2	0.71	20.41	<0.001	0.71	
	C3	0.70	20.34	<0.001	0.66	
Digestive tract	D1	0.61	16.38	<0.001	0.61	
	D2	0.64	19.03	<0.001	0.64	
	D3	0.72	20.25	<0.001	0.72	
Immune system	I1	0.59	16.34	<0.001	0.58	
	I2	0.47	13.70	<0.001	0.47	
	I3	0.55	15.23	<0.001	0.55	

### Determination of cut-off point for Arabic-SHSQ-25

**Figure 3:**
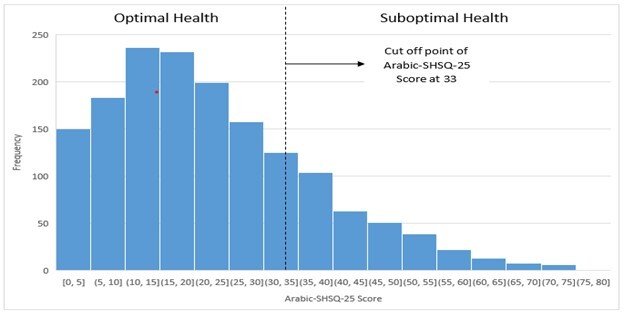
Distribution of Arabic-translated Suboptimal Health Status Questionnaire-25 (ASHSQ-25) scores for the ideal and suboptimal health groups.

Given that the ASHSQ-25 distribution was asymmetrical, the threshold arrived at the one-tailed 90% upper limit of ASHSQ-25 scores [28]; this gave us a cut-off point equal to 33 scores. Thus, an ASHSQ-25 score ≥ 33 represents ‘suboptimal health’, while a score of <33 indicates ‘optimal health’ (**Figure 3**). Therefore, SHS prevalence in this study was 23.7% (377 / 1590).

## DISCUSSION

The current study evaluated the psychometric properties of the Arabic version of the sub-optimal health status questionnaire (ASHSQ-25) in the Saudi Arabian population. Findings supported that ASHSQ-25 has appropriate internal consistency and structural validity; therefore, it is an appropriate tool to measure SHS in Saudi populations. SHS is the physical status between well-being and illness characterised by poorly stated complaints, temporary loss of strength and energy, and a configuration of physical symptoms such as cardiovascular, digestive, immune, and mental status [1,2,24]. Although SHS is not an illness condition, it mainly shows the period antedating the occurrence of clinical symptoms of diseases [1,2]. The SHSQ-25 instrument has earned worldwide recognition for the early detection of chronic diseases in many countries, such as China, Australia, Canada, Ghana, and Russia [10–19]. This instrument is a reliable five domains scale, validated in ethnic groups such as Asian [10,14], Caucasian [17], and African [18]. As an instituted instrument from the view of precision health care, the SHSQ-25 can be applied to discover a person experiencing illness without a diagnosable tool in the health care and community sectors. Therefore, the study aimed to set up an Arabic version of the SHSQ-25 suitable for clinical settings and the health care sector with an Arabic-speaking population.

Following a comprehensive translation process into Arabic language and culture, it has been proven that the ASHSQ-25 is linguistically and conceptually similar to the SHSQ-25 English version. There were no notable problems faced when translating. The ASHSQ-25 retained all questions and structure derived from the SHSQ-25 English version.

This study demonstrates that the ASHSQ-25 has good internal consistency (ICC = 0.92; 95% CI = 0.91 to 0.93) and reliability (Cronbach’s α = 0.92), similar values (ICC = 0.93; 95% CI = 0.91 to 0.95; Cronbach’s α = 0.93) were also observed in the SHSQ-25 English version [12]. Furthermore, the greater response rate of ASHSQ-25 indicates that it is useful among the Arabic-speaking community.

The structural validity of ASHSQ-25 was evaluated using CFA. The Bartlett’s (ᵡ^2^ = 15530.78; *P* < 0.001) and KMO test (0.94) results demonstrate that sampling was adequate to support CFA. The CFA evaluated five domains (i.e. fatigue, cardiovascular system, digestive tract, immune system, and mental health) of ASHSQ-25. The CFA findings displayed a good fit of the databased on the CMIN/df = 4.46, CFI = 0.94, TLI = 0.93, and RMSEA = 0.05, all meeting the appropriate requirements. This study's results demonstrate the consistency of the items in the five domains with the SHSQ-25 English version [10]. The present findings are consistent with earlier investigations that compared the factor structure of SHSQ-25 in Urban Chinese [10], Korea [16], Ghana [18], and, more recently, Iran [14]. This study suggests that the ASHSQ-25 is a valid and reliable instrument for discovering SHS among Arabic-speaking people.

Some limitations should be noted. First, SHS lacks objective clinical diagnosis and is subjective. The data was collected through self-report online methods, which may be influenced by self-reporting. Nevertheless, the large sample size recruited in this current study has contributed to the consistency of the data analysis. Second, the convenience sampling method employed in this study limits the generalisability of the findings. Third, in this study, we employed an online data collection method; thus, the sampling technique limits the participation of those who did not use the internet or information technology. These factors sometimes increase the risk of non-participant error and should be considered while interpreting the findings. However, it is notable that after COVID-19, the number of internet users and mobile applications has significantly increased in Saudi Arabia, as most of the population has had to employ digital tools for various tasks. This is further demonstrated by the fact that in our study, the response rate was 97.4%.

## CONCLUSIONS

In conclusion, our study presents evidence of the construct validity of SHSQ-25 domains in the Arabic-speaking population. The accommodate tools of ASHSQ-25 revealed tolerable internal consistency and construct validity to assess SHS in an Arabic-speaking language population. ASHSQ-25 constitutes an example of defeating language obstacles and alters comparisons when applying the same tool in multiple languages. The ASHSQ-25 is the practicable instrument to monitor and evaluate a person’s health status.

## Additional material


Online Supplementary Document
