# Supplementary material for: Assessing suboptimal health status in the Saudi population: Translation and validation of the SHSQ-25 questionnaire
Source: J Glob Health. 2024 Feb 2;14:04030. doi: 10.7189/jogh.14.04030 (PMC10836270; doi:10.7189/jogh.14.04030)
Supplement: Online Supplementary Document [file jogh-14-04030-s001.pdf]

Appendix S1: ASHSQ-25

تقييم الوضع الصحي دون المستوى الأمثل في الأشهر الثلاثة السابقة. هذه الأسئلة تستفسر عن الأحداث الصحية التي حدثت لك خلال الأشهر الثلاثة الماضية.

الرقم	كم مرة	أبداً أو تقريباً أبداً (0)	أحيانا (1)	غالباً (2)	في اغلب الأحيان (3)	دائماً (4)
1	شعرت بالإرهاق من دون زيادة ملحوظة في نشاطك البدني					
2	شعرت بالإرهاق الذي لا يمكن تخفيفه بأخذ قسط من الراحة					
3	عانيت من الكسل اثناء العمل					
4	عانيت من الصداع					
5	عانيت من الدوخة (الدوار)؟					
6	تألمت عيناك او كانت متعبة					
7	اصبت بالتهاب في الحلق					
8	شعرت بتيبس (تصلب) في العضلات أو المفاصل					
9	عانيت من الم في كتفك او عنقك او خصرك					
10	شعرت بثقل في الارجل عند المشي					
11	شعرت بضيق في التنفس اثناء الجلوس					
12	شعرت باحتقان او ضغط في الصدر					
13	ازعجك خفقان القلب					
14	عانيت من ضعف الشهية					
15	عانيت من حرقة في المعدة					
16	عانيت من الغثيان					
17	لم تستطيع تحمل البيئات الباردة					
18	واجهت صعوبة عند النوم					
19	واجهت صعوبة بسبب الاستيقاظ ليلاً؟ ظللت مستيقظاً طوال الليل					
20	عانيت من مشكلة عدم التذكر الجيد على المدى القصير					
21	عانيت من عدم الاستجابة بسرعة					
22	عانيت من صعوبة في التركيز					
23	تشتت انتباهك بدون سبب					
24	شعرت بالتوتر او كنت عصبي					
25	اصبت بنزلة برد خلال الثلاثة شهور الماضية					
